# Admixture Analysis of Age of Onset in Bipolar Disorder and Impact of Anxiety Comorbidity

**DOI:** 10.7759/cureus.55803

**Published:** 2024-03-08

**Authors:** Stefano Pini, Barbara Carpita, Benedetta Nardi, Marianna Abelli, Giulia Amatori, Ivan Cremone, Liliana Dell'Osso

**Affiliations:** 1 Department of Experimental Medicine, University of Pisa, Pisa, ITA

**Keywords:** psychotic features, anxiety disorders, comorbidity, age of onset, bipolar disorder

## Abstract

Background: The present study aimed to examine clinical differences between subjects with early-onset (<21 years) and adult-onset (>30 years) bipolar I disorder, in particular, in relation to anxiety comorbidity.

Method: Subjects were selected from a cohort of 161 consecutive patients with bipolar disorder type I as diagnosed by the Structured Clinical Interview for DSM Disorder (SCID-I). Clinical characteristics and axis I comorbidity were compared between those whose illness first emerged before the age of 21 years (n=58) and those whose first episode occurred after the age of 30 years (n=27). Psychopathology was assessed using the 18-item version of the Brief Psychiatric Rating Scale (BPRS). The frequency of delusions, hallucinations, and formal thought disorders was evaluated with the SCID-I. Overall, social and occupational functioning was assessed by the Global Assessment of Functioning (GAF).

Results: Most subjects with early-onset bipolar disorder were males, had panic disorder and substance abuse comorbidity, longer duration of illness, exhibited mood-incongruent delusions, and presented with a mixed episode at onset more frequently than the later adult-onset subjects. Mixed mania at the first episode of illness and lifetime panic disorder comorbidity predicted mixed polarity at the first hospitalization episode in the early-onset subjects.

Conclusions: Overall, early age at onset seems to delineate a distinct bipolar I disorder subtype characterized by a greater likelihood of mixed episodes, lifetime panic disorder comorbidity, and schizophrenia-like delusions.

## Introduction

Bipolar disorder (BD) is a chronic mental health condition affecting almost 4% of the population and is characterized by high morbidity and mortality rates. BD has a recurring or chronic course and is linked to psychological impairment and decreased functioning. People with bipolar illness typically go through their first affective episode in their adolescence or early adulthood, although it can take five to 10 years for a diagnosis to be made [1].

It is now known that the age at which BD begins plays a significant role in the progression and prognosis of the condition. Onset in BD is reported to be associated with a longer delay to treatment, greater severity of depression, and higher levels of comorbid anxiety and substance abuse [2]. The age of onset of BD may be a key indicator for identifying homogeneous subtypes in terms of clinical features and comorbidity and ultimately lead to important diagnostic and prognostic implications [3,4]. Several studies carried out in diagnostically different bipolar populations have addressed this issue with inconsistent results. Some of the first studies on the topic were carried out by Carlson et al., who hypothesized that adolescent-onset manic-depressive illness might be a more severe or distinct type of affective disorder [5]. However, when they compared early-onset subjects with those whose age of onset was after 45 years of age, age of onset was not found to be a factor discriminating the two groups on a series of clinical variables. The same authors reappraised this issue 20 years later [6]. By comparing bipolar subjects with adult-onset (after 30 years of age) and early-onset (before 21 years of age), the early-onset group showed more diagnostic instability, higher rates of comorbidity (including childhood psychopathology), greater severity of psychosis, and higher rates of chronicity over a two-year follow-up period. Although the specific clinical features associated with an early-onset have not always been consistent, over past decades numerous studies have highlighted how early-onset BD has been associated with a worse prognosis than late-onset BD, including more psychotic features, drug and alcohol abuse disorders, comorbidity with panic and obsessive-compulsive disorders, rapid cycling, lower lithium-response, and more suicide attempts [7-9]. A recent meta-analysis carried out on 15 papers confirmed the data given from prior studies [10,11], indicating that early-onset bipolar patients have more severe phenomenology, including greater suicidality, higher prevalence of alcohol abuse disorder, twice the prevalence of comorbid substance use disorders, and greater anxious symptoms [2]. Observed correlations with genetic, cognitive, circadian, inflammatory, and brain imaging potential indicators, as well as environmental susceptibility factors including childhood trauma, support the validity of this early age of onset subgroup [2]. Interest in characterizing early onset is also stimulated by the frequent misdiagnosis of BD with psychotic features in young people, presumably because their affective symptoms are either obscured by the severity of their psychosis or mistaken for schizophrenia [1,12,13]. Schurhoff et al. compared patients with early onset (before 18 years) and late-onset (after 40 years) BD and found that the early onset group had the most severe form of the disorder with more psychotic features, higher frequency of mixed episodes, poorer prophylactic lithium response, and higher frequency of comorbid panic disorder (PD) [14]. As to the relation of comorbidity with age at onset, McElroy et al. examined the prevalence and clinical correlates of axis I comorbidity in a cohort of 288 patients with BD type I and II and found that axis I comorbidity was associated with earlier age at onset of BD [15]. In the present study, we aimed to explore the effect of age at onset, especially in relation to anxiety comorbidity. We chose to explore PD, obsessive-compulsive disorder (OCD), and social anxiety disorder (SAD) and observed the effect of these anxiety disorders on early onset and late onset of BD type I. For this purpose, this study made use of admixture analysis to deconstruct the usual non-normal distribution of age at onset in BD. The admixture analysis is a means of estimating the components of a mixture of two or more normal distributions; the combined distribution can be either normal or skewed.

## Materials and methods

The original sample consisted of 165 consecutive out-patients over 16 years old with a Diagnostic and Statistical Manual of Mental Disorder, Fourth Edition (DSM-IV) diagnosis of BD type I; 58 subjects had a most recent manic episode, 68 had a most recent mixed one, and 39 had a depressive one. All subjects were recruited in the context of the University of Pisa Comorbidity in Psychosis Project. Psychotic symptoms secondary to acute intoxication or withdrawal from substances and the presence of a concomitant severe medical condition were considered exclusion criteria. The inclusion diagnosis of BD was made by a senior psychiatrist not directly involved in the study and all patients gave written informed consent to participate. Patients were assessed using the Structured Clinical Interview for DSM Disorders (SCID-I). The SCID-I interviews and psychopathology ratings were performed by resident psychiatrists with at least three years of working experience with bipolar patients and with substantial familiarity with DSM criteria. In addition to the SCID-I patient interview, additional information was obtained from all available sources, i.e., medical records, first-degree relatives, and treating clinicians. The presence of lifetime/current comorbidity for axis I anxiety disorders was also assessed using the SCID. The frequency of delusions, hallucinations, and formal thought disorders was evaluated with the SCID. Inter-rater reliability among the three interviewers was assessed in nine interviews (n=27 interviews). Cohen’s kappa agreement ranged from 0.88 to 1 for diagnosis. The Brief Psychiatric Rating Scale, the Scale for the Assessment of Negative Symptoms, and the Hopkins Symptom Checklist were used to evaluate psychopathology. The Scale to Assess Unawareness of Mental Disorders was used to gauge people's awareness of sickness while the Global Assessment of Functioning Scale (GAF) was employed to assess social and occupational functioning. More detailed information on the methods of this study has been described in previous papers [16].

Statistical analyses

Analytic Procedures for Age at Onset

Admixture analysis was performed for the entire sample of bipolar patients. The "DENORMIX" program, written by Kolenikov and run on the Intercooled Stata 8.2 software (College Station, TX: Stata Corp.) was used to compute the models. The program identifies models composed of multiple normal curves fitting the observed distribution of age at onset. The rationale behind the admixture analysis is that if we can describe a non-normal distribution as composed of multiple normal curves, it is expected that each normal curve describes a more homogeneous sub-group. The assumption underlying such reasoning is that many natural phenomena are approximately normally distributed. A chi-square Pearson goodness of fit test and two types of information criteria (Akaike {AIC} and Schwarz Bayesian Information Criteria {SBIC}) were used to determine the best-fitting models. Intersections of the density curves composing the final models for BD I were adopted to calculate empirical Bayesian cut-offs. Classes were determined by the Bayesian optimal classification rule that puts an individual observation into the class with the highest posterior probability. With this procedure, we obtained subgroups of patients for age at onset and compared them for several sociodemographic and clinical characteristics. This statistical approach has been validated and used in numerous studies [17].

Clinical Comparisons

Data analyses were performed using the SPSS version 20.0 (Armonk, NY: IBM Corp.). Categorical variables were compared by using the chi-square test. Continuous variables were analyzed by using independent sample t-tests. To examine which variables were associated with mixed polarity of index episodes, a stepwise logistic regression was performed. Logistic regression coefficients have been used to estimate odds ratios for each of the independent variables in the model. An alpha level of 0.05 was selected as the cutoff for inclusion in the regression.

## Results

**Figure 1:**
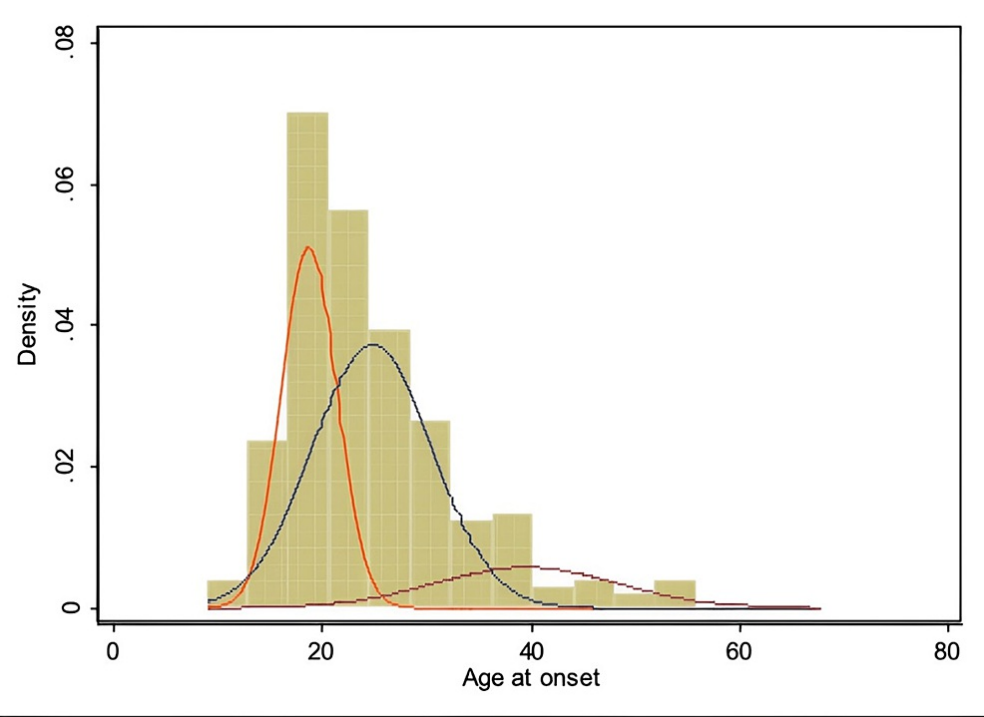
Graphical relation between the empirical histogram and the admixture model with three components.

Of the age of onset models for bipolarity tested with admixture analysis, the one composed of three normal curves best fit the observed overall distribution. Hence, three normally distributed subgroups of age at the onset of bipolar I patients have been identified. Figure 1 shows the distribution for age at onset derived from the admixture analysis of our 161 bipolar I patients.

For this study, within the overall cohort of 161 patients with BD type I, two groups were identified according to the mean age at onset of illness. The first group included subjects whose mean age at onset was earlier than 21 years (n=58), while the second included subjects whose mean age at onset of mood disorder occurred after the age of 30 years (n=27).

Demographic characteristics

As expected, the two groups differed in mean age at the first hospitalization episode (30.8±10.8 vs. 47.2±8.2, t=-6.907, p=0.0001 ). Male subjects were significantly more represented in the early-onset group (50.0% vs. 25.9%) (chi-square=6.060, p=0.014 ). In the early-onset subjects group, never-married subjects were more represented than in the other group (19.0% vs. 44.4%, chi-square=6.060, df=1, p=0.014), this was expected considering the lower mean age in the early-onset group.

Clinical characteristics

Table 1 shows that the age at the onset of BD was earlier, and the length of illness was longer in the early-onset than in the later-onset group. The duration of the last hospitalization did not differ between the two groups.

**Table 1:** Demographic and clinical characteristics of subjects with early-onset (before 21 years of age) and later-onset (after 30 years of age) BD with psychotic features. GAF: Global Assessment of Functioning; BD: bipolar disorder

Characteristics	Early-onset subjects (n=58)	Later-onset subjects (n=27)	t-test	df	p-Value
Age at onset of bipolar disorder	17.42±2.10	38.03±6.57	-1.5906	2.81	<0.001
Number of previous manic episodes	3.17±2.54	2.35±1.66	1.292	2.54	0.202
Number of previous depressive episodes	3.26±3.16	2.18±2.32	1.399	2.58	0.167
Number of hospitalizations	3.03±2.00	2.81±2.02	0.407	2.83	0.640
Length of index hospitalization (days)	31.3±20.7	25.8±11.1	1.500	2.83	0.138
Length of illness	13.10±10.40	9.11±5.98	2.212	2.81	0.030
Level of functioning in the last month (GAF)	30.37±11.49	27.96±13.54	0.819	2.75	0.415

Table 2 shows that psychotic mixed mania, as the reason for hospitalization at the index episode, was significantly more frequent in the early-onset group, while depression as the reason for hospitalization and as the index episode, was significantly more frequent in the later-onset group. The percentage of patients who underwent electroconvulsive therapy during the last episode of illness was higher in the early-onset group, with this difference being nearly statistically significant. Table 3 displays the rates of lifetime and current anxiety and substance abuse axis I disorders in the two groups. The early-onset subjects were significantly more likely to have a comorbid lifetime diagnosis of PD and substance use disorder.

**Table 2:** First episode characteristics and reason for hospitalization. ^a^Three missing cases in the early-onset group and three in the late-onset group. ECT: electroconvulsive therapy

Characteristics	Early-onset subjects (n=58) n (%)	Later-onset subjects (n=27) n (%)	Chi-square	p-Value
Polarity of first affective episode^a^				
Mixed mania	20 (36.4%)	5 (20.8%)	1.863	0.172
Depression	24 (43.6%)	12 (50.0%)	0.273	0.601
Mania	11 (20.0%)	7 (29.2%)	0.798	0.372
Reason for first hospitalization^a^				
Mixed mania	23 (41.8%)	4 (16.7%)	4.699	0.030
Depression	10 (18.2%)	10 (41.7%)	4.874	0.020
Mania	10 (18.2%)	8 (33.3%)	2.180	0.140
Others	12 (21.8%)	2 (8.3%)	2.084	0.149
ECT during the last episode of illness^a^	20 (36.4%)	4 (16.7%)	4.363	0.113

**Table 3:** Lifetime axis I comorbidity in early-onset and adult-onset bipolar patients. OCD: obsessive-compulsive disorder

Lifetime axis I comorbidity	Early-onset subjects	Late-onset subjects	Chi-square	p-Value
Panic disorder	27 (46.6%)	5 (18.5%)	6.168	0.01
Social anxiety disorder	10 (17.2%)	3 (11.1%)	0.534	0.46
OCD	8 (13.8%)	5 (18.5%)	0.318	0.57
Anorexia	3 (5.2%)	0	1.448	0.23
Bulimia	1 (1.7%)	0	0.471	0.49
Substance abuse	18 (31.0%)	2 (7.4%)	5.716	0.01

In Table 4 mean ages of onset of comorbid axis I disorders are presented. Only mean age at the onset of SAD clearly antedate the onset of mood disorder in the two groups. Although the age of onset of PD was significantly earlier in the early-onset group than in the adult-onset group, it did neither appear to antedate the onset of mood disorder in either group nor did the ages at onset of OCD and substance abuse.

**Table 4:** Age at onset of lifetime axis I comorbidity in early-onset and adult-onset bipolar patients. OCD: obsessive-compulsive disorder

Lifetime axis I comorbidity	Early-onset subjects (n=58)	Late-onset subjects (n=27)	t-test	df	p-Value
Panic disorder	20.68±6.77	32.57±11.41	-2.643	2.33	0.033
Social anxiety disorder	10.33±3.75	13.40±2.07	-2.151	2.15	0.050
OCD	18.8±3.22	28.25±18.73	-1.003	2.12	0.388
Anorexia	14.33±6.11	-	-	-	-
Bulimia	19.00±1.41	-	-	-	-
Substance abuse	17.40±1.64	26.00±5.66	-2.138	2.15	0.27

Illness severity

Considering the frequency of psychotic symptoms, as shown in Table 5, early-onset subjects were significantly more likely than later-onset subjects to report broadcasting delusions. Two separate logistic regression analyses of variables predictive of psychotic mixed state at the first hospitalization were performed in the two groups. In Table 6, results obtained in the early-onset group are displayed.

**Table 5:** Psychotic symptoms in early-onset and adult-onset bipolar patients. Probability multiplied by six to correct for multiple comparisons (Bonferroni’s inequality correction). SCID: Structured Clinical Interview for DSM Disorders

SCID delusions	Early-onset subjects (n=58)	Late-onset subjects (n=27)	Chi-square	df	p-Value
Reference	49 (84.5%)	18 (66.7%)	3.503	1	0.061
Persecutory	44 (75.9%)	21 (77.8%)	0.038	1	0.846
Grandiosity	32 (55.2%)	12 (44.4%)	0.849	1	0.357
Other	11 (19.0%)	8 (29.6%)	1.207	1	0.272
Control	23 (39.7%)	5 (18.5%)	3.726	1	0.054
Systematized	21 (36.2%)	15 (55.6%)	2.825	1	0.093
Bizarre	16 (27.6%)	3 (11.1%)	2.881	1	0.090
Broadcasting	15 (25.9%)	1 (3.7%)	5.920	1	0.015
Somatic	8 (13.8%)	4 (14.8%)	0.016	1	0.900

**Table 6:** Results of logistic regression analysis in the early-onset group. Logistic regression with mixed polarity at index episode as dependent variable and comorbidity, lifetime panic disorder, and mixed polarity at first episode as independent variables. Exp(B): exponentiated B-coefficients; Sig.: statistical significance

Variables	B	SE	Wald test	df	Sig.	Exp (B)	95.0% CI for exp (B)	
							Lower	Upper
Mixed polarity in first episode	2.302	0.949	5.890	1	0.015	9.998	1.558	64.185
Lifetime panic disorder	1.792	0.876	4.182	1	0.041	5.999	1.078	33.371
comorbidity	-2.262	1.310	2.980	1	0.084	0.104	0.008	1.358
Constant	-1.609	0.632	6.475	1	0.011	0.200	-	-

Lifetime PD comorbidity and a mixed state polarity of first episode of illness were found to be significantly associated with a psychotic mixed state at first hospitalization. Such an association was not found in the adult-onset group.

## Discussion

In a cohort of patients with BD type I, early-onset and adult-onset disorders were found to have important phenomenological differences. First, compared to the later-onset group, early-onset subjects exhibited more frequently a psychotic mixed state with mood incongruent delusions at the first hospitalization episode. Furthermore, in the early-onset group, but not in the later-onset group, logistic regression analysis showed that index mixed episode was predicted by a mixed first episode of illness. Numerous studies indicate that the course of bipolar illness is predominantly characterized by episodes of the same polarity as the first one [16,18,19]. Accordingly, our data suggest that the polarity of recurrences was likely to be predominantly mixed in this younger group of bipolar patients, often implying difficulties in therapeutical management.

Second, consistent with previous studies reporting higher rates of mood incongruent features and first-rank symptoms [19,20], greater likelihood of schizophrenia spectrum diagnosis [21], or more florid psychotic symptoms among early-onset bipolar patients, we found that early-onset bipolar patients were characterized by a higher frequency of broadcasting delusions at first hospitalization than in the adult-onset group [22]. This factor probably adds to the diagnostic complexity and confusion in these patients, particularly when the expansive-excited component loses intensity and thought disturbances emerge quite openly, often resulting in a misdiagnosis of schizophrenia. In this framework, recent studies have reported that around 16% of patients with a final diagnosis of BD had previously received a diagnosis of a non-affective psychotic disorder. Due to a vast symptomatologic overlap between the two conditions, it can be challenging to accurately diagnose subjects who share manic or depressive mood and psychotic manifestations into these two diagnostic categories. Indeed, a considerable proportion of patients with schizophrenia may experience manic or depressed episodes either before or during psychotic episodes, whereas individuals with BD may experience psychotic symptoms during manic or depressive episodes. Furthermore, common etiological factors for these two disorders have been largely reported, such as infectious agents, drug use, obstetric complications, and environmental and genetic factors [22]. Therefore, early identification between the two disorders greatly influences the pharmacologic and psychological therapy regimens, as well as the prognoses. It is crucial for effective treatment, as a timely initiation of mood stabilizers in BP subjects is correlated with a higher response and better outcome. In this regard, future research is warranted to explore the interaction between depressive and manic components of mixed episodes, which prevail in younger patients, and the development of schizophrenia-like delusions.

Third, lifetime PD comorbidity has been found to be significantly more frequent in the early-onset than in the later-onset group. These data are consistent with the few previous studies that explored the association between axis I anxiety comorbidity and an earlier age at onset of BD [14,15,17]. The relation between PD and mixed state in the early-onset group was also found by Schurhoff et al. in a sample of 210 bipolar I and II patients [14]. Further studies should explore whether specific anxiety disorders might be determinants for the genesis of mixed states in these patients. The chronological relation between the onset of PD and BD was less clear. Paralleling age at onset of mood disorder, age at onset of PD was significantly earlier in the early-onset bipolar group than in the later-onset group. However, while comorbid SAD clearly predated at least three years of the onset of BD in almost all cases, for bipolar subjects with comorbid PD, the onsets of the two disorders did not show a clear chronological relationship. According to their mean age at onset, in the early-onset group, PD onset seemed to follow that of mood disorder, while in the later-onset group, the result was in the opposite direction. The literature on the chronology of comorbid and principal mood syndrome in psychotic bipolar patients is quite limited. Strakowski et al. found that, in 73% of cases, anxiety comorbidity was antecedent to BD with psychotic features by at least one year [23]. In this study; however, PD comorbidity was not specifically investigated. Similarly, a recent systematic review highlighted early-onset panic attacks and PD, as well as SAS, as clinical risk factors for BD. Our data highlight the need for further studies on the chronology of PD and BD when the two conditions co-occur and clarify whether panic comorbidity may be a prodromal syndrome or a factor complicating bipolar illness. The comorbidity with PD has been proposed to be a marker of genetic heterogeneity of BD. The fact that we found high comorbidity with PD in the early-onset group supports the importance of this specifier to identify distinct subtypes of BD.

Substance abuse has been found to occur at higher rates in subjects with early-onset BD. This association has been found in several previous studies conducted in different populations of bipolar patients with or without psychotic features. It will be interesting to explore whether early-onset subjects at high risk for BD have higher rates of mood instability that make them vulnerable to developing BD, especially when persistently abusing substances at a very young age [24].

Male gender has been found to be significantly more represented in the early-onset group. This finding is consistent with previous studies, which found that the male gender is prominent in younger patients with severe bipolar illness in contrast to the usually cited 1:1 gender ratio [25]. In addition, young subjects with early-onset BD (before the age of 21 years) are more likely to have more severe psychopathology (PD comorbidity, mixed episodes, incongruent delusions, and longer duration of illness). These results are largely consistent and corroborate those of some previous studies [7-9,26-29].

Several limitations to the design of this study should be acknowledged. The small sample size and the fact that we studied only hospitalized patients limits the generalizability of our results to patients with BD type I. Moreover, the cross-sectional design of the study prevents us from making inferences about the temporal or causal relationships between the variables. For most comparisons, we did not have sufficient statistical power to analyze separately patients with prevalent manic episodes as compared to those with prevalent mixed or depressive episodes. Furthermore, although systematic interviews of patients and relatives were conducted by skilled clinicians, the study was based on retrospective data collection rather than prospective follow-up.

## Conclusions

In conclusion, our results highlighted significant phenomenological differences between early-onset and adult-onset BD type I disorder. In particular, the early-onset subjects reported higher rates of mood incongruent features, greater likelihood of schizophrenia spectrum diagnosis, more florid psychotic symptoms, and higher PD and substance abuse comorbidity. Moreover, in the early-onset group, the index mixed episode was found to be predicted by a mixed first episode of illness, suggesting a predominantly mixed polarity of recurrences and implying greater difficulties in therapeutical management. Ultimately, the main implication of this study is that it gave further support to the body of evidence indicating that, in patients with BD with psychotic features, age at onset may help to identify subjects with different phenomenological and genetic characteristics and, consequently, critical prognostic factors.
